# Systemic Hydrocortisone To Prevent Bronchopulmonary Dysplasia in preterm infants (the SToP-BPD study): statistical analysis plan

**DOI:** 10.1186/s13063-018-2505-y

**Published:** 2018-03-09

**Authors:** Wes Onland, Maruschka P. Merkus, Debbie H. Nuytemans, Marijke C. Jansen-van der Weide, Rebecca Holman, Anton H. van Kaam, on behalf of the SToP-BPD study group

**Affiliations:** 1 Department of Neonatology, Emma Children’s Hospital, Academic Medical Centre, Room H3-145, PO Box 22700, 1100 DD Amsterdam, The Netherlands; 2 Clinical Research Unit, Academic Medical Centre, Amsterdam, The Netherlands; 3 Paediatric Clinical Research Office, Emma Children’s Hospital, Academic Medical Centre, Amsterdam, The Netherlands

**Keywords:** Hydrocortisone, Preterm, Mortality, Bronchopulmonary dysplasia, Statistical analysis plan

## Abstract

**Background:**

Bronchopulmonary dysplasia (BPD) is the most common complication of preterm birth with short-term and long-term adverse consequences. Although the glucocorticoid dexamethasone has been proven to be beneficial for the prevention of BPD, there are concerns about an increased risk of adverse neurodevelopmental outcome. Hydrocortisone has been suggested as an alternative therapy. The aim of the Systemic Hydrocortisone To Prevent Bronchopulmonary Dysplasia in preterm infants (SToP-BPD) trial is to assess the efficacy and safety of postnatal hydrocortisone administration for the reduction of death or BPD in ventilator-dependent preterm infants.

**Methods/design:**

The SToP-BPD study is a multicentre, double-blind, placebo-controlled hydrocortisone trial in preterm infants at risk for BPD. After parental informed consent is obtained, ventilator-dependent infants are randomly allocated to hydrocortisone or placebo treatment during a 22-day period. The primary outcome measure is the composite outcome of death or BPD at 36 weeks postmenstrual age. Secondary outcomes are short-term effects on pulmonary condition and long-term neurodevelopmental sequelae assessed at 2 years corrected age. Complications of treatment, other serious adverse events and suspected unexpected serious adverse reactions are reported as safety outcomes. This pre-specified statistical analysis plan was written and submitted without knowledge of the unblinded data.

**Trial registration:**

Netherlands Trial Register, NTR2768. Registered on 17 February 2011. EudraCT, 2010-023777-19. Registered on 2 November 2010.

## Background

Bronchopulmonary dysplasia (BPD) is the most common complication of preterm birth [1–3]. Pulmonary inflammation has been identified as an important risk factor in the development of BPD, providing the rationale for treating patients at risk for BPD with glucocorticoids, a group of drugs well known for their strong anti-inflammatory effect [4]. Randomised controlled trials (RCTs) comparing dexamethasone with placebo in ventilated preterm infants at risk for BPD have shown that dexamethasone reduces the incidence of the composite outcome of death or BPD [5, 6]. Unfortunately, follow-up studies showed associations with adverse neurodevelopmental outcome [5]. Therefore, international guidelines have stated that the use of dexamethasone should be restricted to exceptional cases (i.e., patients who cannot be weaned off the ventilator), and clinical trials should be performed to investigate the efficacy and safety of alternative anti-inflammatory glucocorticoids such as hydrocortisone (HC) to prevent BPD [7, 8].

The Systemic Hydrocortisone To Prevent Bronchopulmonary Dysplasia in preterm infants (SToP-BPD) study is the first multicentre, placebo-controlled, double-blind RCT investigating the efficacy and safety of systemic HC initiated between 7 and 14 days after birth in mechanically ventilated preterm infants. The study protocol was published previously [9]. This paper describes the final statistical analysis plan (SAP) in detail. This SAP was written and submitted without knowledge of the unblinded data.

### Objectives

The primary aim of the SToP-BPD trial is to investigate if systemic HC started between 7 and 14 days after birth is effective and safe in reducing the incidence of the composite outcome of death or BPD at 36 weeks postmenstrual age (PMA) in chronically ventilated preterm infants, as compared with a placebo (primary endpoint).

## Methods/design

### Design and setting

The SToP-BPD study is a multicentre, phase III, placebo-controlled, double-blind superiority trial in mechanically ventilated preterm infants at risk for BPD, performed in 19 neonatal intensive care units (NICUs) in the Netherlands and Belgium.

#### Study protocol development and conduct

The SToP-BPD study is registered with the Netherlands Trial Register (NTR2768; registered on 17 February 2011) and the European Union Clinical Trials Register (EudraCT, 2010-023777-19; registered on 2 November 2010). The ethics committee of the Academic Medical Centre in Amsterdam approved the study protocol on 28 January 2011, starting with ten Dutch and five Belgian NICUs. Four Belgian NICUs joined the study at a later date. The local ethics committee of each participating hospital approved the local feasibility of the study protocol. During the course of the study, the accredited ethics committee approved two amendments regarding changes of the inclusion criteria (*see below*). The trial was conducted according to the principles of the Declaration of Helsinki, Dutch and Belgium legislation regarding medical research involving human subjects [10–13], and good clinical practice (GCP) guidelines [14]. Patients could be included in the trial only after written informed consent from both parents was obtained. All study sites were monitored by an independent clinical research associate of the Academic Medical Centre Clinical Research Unit (Amsterdam, the Netherlands). An independent data and safety monitoring board (DSMB) monitored the study’s progress, with a special focus on safety (*see below*). The trial will be reported according to the Consolidated Standards of Reporting Trials (CONSORT) guidelines [15].

Inclusion and exclusion criteria are described in the published study protocol [9]. Ventilator-dependent preterm infants (gestational age < 30 weeks and/or birth weight < 1250 g) at high risk of developing BPD were included between 7 and 14 days postnatal age (PNA). The risk of developing BPD was assessed by the respiratory index (RI), defined as the product of mean airway pressure (MAwP) and the fraction of inspired oxygen (FiO_2_). In the absence of firm evidence, a high risk of developing BPD was arbitrarily defined as an RI ≥ 3.5 for > 12 h/day for ≥ 48 h, ensuring adequate oxygen saturation (85–95%) and partial pressure of carbon dioxide values (5.0–7.5 kPa). However, during the first months of the trial, the participating centres found that this arbitrarily chosen RI cut-off of 3.5 was too high. The centres noted that many infants ventilated between 7 and 14 days PNA with an RI < 3.5 were still at high risk of developing BPD, as indicated by the fact that most of these infants were treated with corticosteroids outside the trial. Therefore, the RI threshold in the inclusion criteria was reduced stepwise, first to 3.0 (21 May 2012) and finally to 2.5 (3 December 2012), to make sure that all infants at high risk of BPD could be included in the study. Both RI adjustments were submitted as protocol amendments to the accredited ethics committee and approved.

Included participants were allocated to either HC or placebo treatment according to the study protocol. In general, the use of open-label HC during the 22-day treatment course was strongly discouraged. However, open-label HC use was allowed if the patient met the predefined criteria described in the study protocol [9]. The open-label HC dosage schedule was similar to that used in the study. The study medication was stopped when open-label HC was initiated. Patients receiving open-label HC were followed in the same way as all other patients.

#### Randomisation and data collection

Eligible patients were randomly allocated in a 1:1 ratio to either HC or placebo, stratified by centre and gestational age [9]. To ensure allocation concealment, the randomisation sequence was generated by using fully GCP- and U.S. Food and Drug Administration-compliant ALEA® randomisation software (www.formsvision.com; ALEA Clinical/FormsVision, Abcoude, the Netherlands). Randomisation was centrally controlled and web-based, using a dedicated, password-protected, SSL-encrypted website. The infants’ parents, all members of the research team, and the medical team remained blinded to group assignment throughout the study. Data management was implemented according to GCP guidelines. Patient data until hospital discharge and long-term follow-up data are entered via an electronic case record form in a central GCP proof web-based database to facilitate on-site data entry (Oracle® Database Reference 12c Release 1 [12.1]; Oracle, Redwood Shores, CA, USA), as described in the study protocol [9]. Security is guaranteed with login names, login codes and encrypted data transfer.

#### Primary outcome

The primary outcome is the dichotomous composite outcome of death or BPD at 36 weeks PMA (BPD-free survival). Patients were categorised as having BPD when needing supplemental oxygen and/or positive pressure support at 36 weeks PMA, assessed according to the Eunice Kennedy Shriver National Institute of Child Health and Human Development Consensus Statement using the classification of severity as proposed by Jobe et al. [4] and, if indicated, the oxygen reduction test as described by Walsh et al. [16, 17].

#### Secondary outcomes

##### Study phase I: short-term outcomes before initial hospital discharge

Secondary efficacy outcomes assessed during the study phase before initial hospital discharge include mortality (at 28 days PNA, 36 weeks PMA and hospital discharge), BPD (at 28 days PNA and 36 weeks PMA, including severity grade), failure to extubate (at 3, 7, 14 and 21 days after initiating study medication), total duration of mechanical ventilation, total time receiving supplemental oxygen, use of open-label HC treatment, hospital length of stay, necrotising enterocolitis (NEC) with Bell stage II or higher [18], gastrointestinal bleeding, spontaneous intestinal perforation (SIP), intraventricular haemorrhage (IVH) and/or periventricular leucomalacia (PVL) [19], retinopathy of prematurity (ROP) [20], hypertension, hyperglycaemia requiring the use of insulin therapy, nosocomial infection including clinical or culture-proven sepsis, meningitis and pneumonia, patent ductus arteriosus for which medical intervention or surgical ligation is needed, and growth (weight, head circumference, and length at 36 weeks PMA).

##### Study phase II: long-term follow-up outcomes at 2 years corrected age

Neurodevelopmental impairment at 2 years corrected age (CA) will be assessed according to the cognitive and motor composite scores of the Bayley Scales of Infant and Toddler Development, Third Edition (BSID III) [21]. Norm value of BSID III scores is 100 (SD 15), with higher values indicating better function. Other secondary outcomes are: mortality, number of hospital readmissions since first discharge home, cerebral palsy and its severity assessed by the gross motor function classification system [22], hearing loss requiring hearing aids, blindness, behavioural problems (Child Behavior Checklist [23]), weight, length and head circumference.

#### Safety

Safety outcomes were classified as follows:

1. Complications of HC treatment (*see* “Secondary outcomes: Study phase I: short-term outcomes before initial hospital discharge” subsection above);
2. Suspected unexpected serious adverse drug reactions (SUSARs); and
3. Other serious adverse events (SAEs).

### Statistical methods specified in the study protocol

#### Sample size calculation

As described in the study protocol, the a priori risk of death or BPD in preterm infants < 30 weeks gestation and ventilated in the second week of life is estimated at 60–70% [9]. Authors of a meta-analysis of moderately early dexamethasone treatment estimated an absolute risk reduction (ARR) of 25% (number needed to treat [NNT] = 4) compared with placebo [24]. No data are currently available on the efficacy of HC, and the suggested cumulative dose in the present study is considerably lower than previously used dexamethasone doses. Because the efficacy of dexamethasone is dependent on the doses used [25, 26], we proposed a more conservative approach, defining an ARR of ≥ 15% (NNT = 7) as clinically relevant and still approaching the reported efficacy of dexamethasone.

If the probability of death or BPD at 36 weeks PMA is 0.60 in the placebo arm and 0.45 in the HC group, then including 175 patients in each group (total 350, chi-square test without continuity correction) and a two-tailed type I error of 0.05 will result in a power of 80% (Statistical Solutions Ltd., Cork, Ireland). We anticipated that ~ 10% of patients would drop out of the study and hence aimed to include 200 patients in each treatment arm (total 400).

#### Originally proposed analyses

In the previously published protocol [9], the originally proposed analyses are described, including both an intention-to-treat (ITT) and per-protocol (PP) analysis because use of open-label glucocorticoids may modulate possible treatment effects. The effect of HC on the primary outcome of BPD-free survival is assessed by multivariable logistic regression analysis including possible confounders. *p* Values < 0.05 are viewed as statistically significant. Below we present the final and further detailed SAP.

#### Interim analyses and safety reporting

A DSMB was installed for this study to protect patients and to assist and advise the principal investigator in protecting the safety, validity and credibility of the trial. Members included a clinically experienced paediatric pulmonologist, a paediatric intensivist-clinical pharmacologist with extensive knowledge of the use of drugs in infants, and a biostatistician. The members were not involved in the trial and had no competing interests. The composition, tasks, responsibilities and working procedures of the DSMB were described in a charter.

In the first 2 years of the study, interim analyses for safety issues were performed every 3 months. After this period, the frequency of safety monitoring was reduced to thrice and then twice annually, owing to the decreasing recruitment rate. The DSMB received a report before every interim analysis prepared by the trial statistician that included data on baseline variables (gestational age, birth weight, sex, multiple births), the primary outcome parameter, predefined safety outcomes, and SAEs and SUSARs. In the closed session, attended only by the DSMB members, the DSMB examined the data using coded (blinded) treatment groups. If necessary, treatment allocation could be unblinded.

The DSMB charter stated that there were two possible reasons for stopping the study early, namely concerns for safety and futility. In principle, the trial was not to be stopped early for beneficial effect of HC treatment on the primary outcome before the minimum number of evaluable patients required (350) was included. This was done to preserve the power for evaluation of neurodevelopmental outcome at 2 years CA, unless HC benefit was accompanied by an unacceptably high rate of mortality in the placebo group. Hence, there was no alpha spending associated with the interim analyses.

Regarding safety, the study would be stopped at an interim analysis if one or more of the following safety issues occurred and a two-sided *p* value < 0.10 (blinded analyses) or a one-sided *p* value < 0.05 (unblinded analyses) was obtained:

- Mortality was ≥ 10% higher in the HC group than in the placebo group
- Combined NEC Bell stage III and SIP ≥ 10% higher in the HC group than in the placebo group
- ROP stage III or higher ≥ 10% higher in the HC group than in the placebo group
- PVL stage I or higher ≥ 5% higher in the HC group than in the placebo group

In addition, the study would be stopped following 50% or 75% of planned inclusion if the primary outcome (composite endpoint of mortality or BPD at 36 weeks PMA) was ≥ 15% higher owing to ≥ 10% higher mortality in the placebo group than in the HC group. If the difference in the primary outcome between the placebo and HC groups was < 10% following 75% of the planned inclusion, the trial was to be stopped for futility. All differences in percentages between the groups defined in the DSMB charter were absolute differences. If one or more of these situations occurred, the clinical relevance, reliability and consistency of the results were incorporated into the decision whether to end the trial prematurely.

### Statistical analysis plan

#### Overall principles

The first phase of data analysis and reporting will include all outcome data up to discharge to home. Analysis will start once all data to discharge of the last included patient have been obtained, the database has been cleaned and locked, and the SAP has been submitted for publication. The analyses will be performed by a statistician and methodologist, neither of whom is involved in the assessment of trial outcomes.

Analyses will be performed according to the ITT principle. Given the modulating effects of open-label corticosteroids [27], analyses will also be done in an as-treated (AT) population and a PP population (*see below*) to check the robustness of the main analyses. For all relevant parameters, 95% CIs will be presented. *p* Values < 0.05 will be regarded as statistically significant. For statistical programming and analysis, we will use the IBM SPSS Statistics version 24 statistical software package (IBM, Armonk, NY, USA) and the R environment for statistical computing (R Foundation for Statistical Computing, Vienna, Austria).

The statistician and methodologist will perform the analysis blinded to the allocated study treatment and using a fictive randomisation code. After the data analyses have been completed, they will be repeated using the true treatment allocation. These analyses will be performed and published before the assessment of long-term outcomes at 2 years CA of all included patients have been completed. As in other trials in neonatology, it is deemed unethical to withhold the results of the primary outcome analyses from the international community for another 2 years.

This first phase of data analysis and reporting will be followed by a second phase, analysing and reporting the long-term neurodevelopmental outcomes after 2 years. Clinicians involved in care during the initial hospitalisation, outcome assessors of the neurodevelopmental outcome at 2 years CA, and parents will be kept blinded to the treatment allocation of all included patients until this second report has been published.

#### Handling of missing data

In case of missing data, every attempt will be undertaken to retrieve the data. We will contact both levels III and II (referral) hospitals because many infants will be transferred back to referral hospitals once clinically stable [4, 28]. Because the primary outcome will be assessed before hospital discharge, we anticipate no or minimal missing values. In case an oxygen reduction test is indicated but has not been performed, a committee of three independent experts will assess the severity of the BPD diagnosis. These experts will be kept blinded to the allocation arm during this assessment. Owing to frequent standard care follow-up visits and strong relationships between physicians and the families of the preterm infants, we anticipate little missing follow-up data. All cases lost to follow-up will be recorded, including their available key characteristics and trial results and reason for the loss to follow-up. Missing data will not be imputed, with the exception of the long-term neurodevelopmental outcomes [*see* “Long-term outcomes at 2 years corrected age (study phase II)” section below.

#### Definition of analysis sets

##### Intention-to-treat population

The ITT population includes all randomised infants, regardless of protocol deviations (*see below*) or use of open-label corticosteroids (Table 1). This includes patients with a signed informed consent for the study who were randomised but died before receiving the first dose of study medication.

**Table 1:** Definition of population analysis sets

Analysis population	HC treatment group	Placebo treatment group
Intention to treat ‘as randomised’	*HC randomisation*: • Including all protocol deviations (e.g., in eligibility criteria)	*Placebo randomisation*: • Including all protocol deviations (e.g., in eligibility criteria)
As treated ‘actual treatment’	*Patient received HC (at least one dose), regardless of allocated treatment at randomisation:* • Including those infants who received rescue open-label corticosteroids as described according to protocol • Including those infants who received rescue open-label corticosteroids not following protocol (protocol deviation) • Including those infants with other protocol deviations (e.g., in eligibility criteria)	*Patient received placebo (at least one dose), regardless of allocated treatment at randomisation:* • Including those infants not receiving any corticosteroid dose for pulmonary reasons • Excluding those infants who received rescue open-label corticosteroids following study protocol • Excluding those infants who received rescue open-label corticosteroids not following study protocol • Including those infants with other protocol deviations (e.g., in eligibility criteria)
Per protocol	*HC randomisation and treated according to study protocol*: • Including those infants receiving rescue open-label corticosteroids as described by the study protocol • Excluding those infants receiving rescue open-label corticosteroids not according to study protocol (protocol deviation) • Excluding other protocol deviations (e.g., in eligibility criteria)	*Placebo randomisation and treated according to study protocol*: • Including those infants receiving rescue open-label corticosteroids following study protocol • Excluding those infants receiving rescue open-label corticosteroids not following study protocol (protocol deviation) • Excluding other protocol deviations (e.g., in eligibility criteria)

*HC* hydrocortisone

##### As-treated population

Infants will be analysed in groups according to the actual treatment received (i.e., yes or no corticosteroids), regardless of allocated treatment at randomisation (Table 1). Infants will still be included in the AT population if there was a protocol deviation (*see below*).

##### Per-protocol population

In the PP population, all patients included and treated in accordance with the study protocol will be included (Table 1). Patients allocated to the placebo group but treated with open-label corticosteroid treatment in compliance with the study protocol will be included in the PP placebo group. Infants in both treatment groups treated with open-label corticosteroids outside the study protocol or in whom other protocol deviations (*see below*) occurred will be excluded from the PP population.

### Statistical analyses

#### Patient flow

**Fig. 1:**
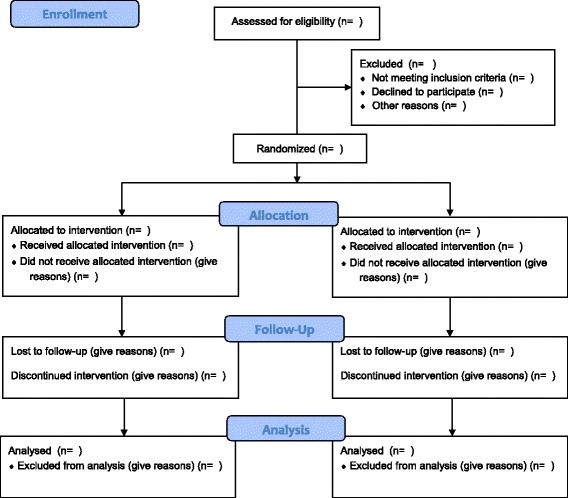
Consolidated Standards of Reporting Trials (CONSORT) 2010 flow diagram

The flow of participants is displayed in the CONSORT flow diagram (Fig. 1). Reasons that eligible patients were not included, such as parents declined consent or the medical team did not ask the parents, will be summarised.

#### Protocol deviations

Protocol deviations are defined as deviations in eligibility criteria and open-label corticosteroid treatment not conforming to conditions set forth in the study protocol, or unblinding. All protocol deviations will be line-listed.

#### Baseline characteristics

We will present the following maternal characteristics: age, ethnic origin, highest completed level of education, mode of delivery, antenatal corticosteroids, chorioamnionitis, pre-eclampsia and multiple pregnancy. We will also present the following baseline characteristics of patients: gestational age; sex; birth weight; small for gestational age; Apgar score at 5 minutes; surfactant therapy; pulmonary air leak syndrome; patent ductus arteriosus; clinical and culture-proven sepsis before randomisation; inhalation of nitric oxide and hypotension, NEC, SIP and/or IVH before randomisation; age at randomisation; and FiO_2_ MAwP at start of study medication. All variables will be presented as summary statistics according to allocation arm of the trial.

Continuous normally distributed variables will be summarised using the mean and SD. Continuous non-normally distributed variables will be summarised using the median and IQR. Categorical variables will be summarised using counts and percentages. No formal testing will be performed on the baseline characteristics.

#### Short-term outcomes until initial hospital discharge (study phase I)

##### Primary outcome

In Table 2, an overview is given of all study outcomes with their planned analysis methods. Crude estimates of the relative risk and ARR of the primary outcome in the HC group compared with the placebo group will be calculated. The main analysis of the effect of HC on the primary outcome will be performed using a logistic regression model, correcting for the stratification factors gestational age (less than or greater than or equal to 27 weeks) and centre. Adjusting for stratification by centre can lead to problems with the convergence or stability of estimates of statistical parameters if some or all centres are small [29]. To ensure that the estimates of the regression parameters converge, hospitals with fewer than ten subjects without and/or fewer than ten subjects with the composite endpoint BPD-free survival at 36 weeks PMA will be combined in this analysis [30]. The effect sizes from this model will be expressed as an adjusted OR.

**Table 2:** Variables/outcomes to be presented and planned statistical analysis methods

Variable/outcome	Type of outcome	Statistical analysis
*Study phase I: short-term outcomes until initial hospital discharge*		
BPD-free survival at 36 weeks PMA	Primary	Logistic regression with correction for stratification factors plus sensitivity and sub-group analyses
Death at 28 days PNA, at 36 weeks PMA and at hospital discharge BPD at 36 weeks PMA Failure to extubate 3, 7, 14 and 21 days after start of trial medication Time to extubation, censored at death Time to supplemental oxygen independence Time to hospital discharge Necrotising enterocolitis Gastrointestinal bleeding Spontaneous intestinal perforation Intraventricular haemorrhage and/or periventricular leucomalacia Retinopathy of prematurity Hypertension Hyperglycaemia requiring insulin therapy Nosocomial infection, including clinical or culture-proven sepsis, meningitis, pneumonia Patent ductus arteriosus needing medical intervention or surgical ligation Weight, head circumference, length at 36 weeks PMA Use of open-label HC treatment	Short-term secondary	Linear, logistic or Cox regression or competing risk model, as appropriate
SUSARs, SAEs	Short-term secondary	Descriptive statistics
*Study phase II: long-term follow-up at 2 years CA*		
Survival without neurodevelopmental impairment	Key long-term secondary	Logistic regression with correction for stratification factors, sensitivity and sub-group analyses
Mortality Number of hospital readmissions since first discharge to home Cerebral palsy and its severity Hearing loss requiring hearing aids Blindness Behaviour problems (Child Behavior Checklist)Growth (weight, length, head circumference)	Long-term secondary	Linear, logistic or Cox regression or competing risk model, as appropriate

*Abbreviations: BPD* Bronchopulmonary dysplasia, *CA* Corrected age, *HC* Hydrocortisone, *PMA* Postmenstrual age, *PNA* Postnatal age, *SAE* Serious adverse event, *SUSAR* Suspected unexpected serious adverse drug reaction

In an additional analysis, the effect of HC on the primary outcome will be evaluated using a multivariate logistic regression model including five important biologically plausible baseline risk factors for BPD, pre-selected on the basis of literature and our clinical experience: (a) gestational age (less than or greater than or equal to 27 weeks) [31], (b) the presence of chorioamnionitis [32], (c) RI at randomisation [33], (d) sex [34] and (e) multiple pregnancy [35].

##### Sub-group analyses

We will perform pre-specified exploratory sub-group analyses for the primary outcome by examining treatment × sub-group interaction effects in logistic regression models. We will perform six analyses, each examining one sub-group: gestational age groups (less than or greater or equal to 27 weeks), presence of chorioamnionitis (yes/no), RI at randomisation, sex (male/female), multiple pregnancy (yes/no) and centre preference for type of steroid treatment prior to the study (‘HC’ versus ‘dexamethasone’). Each of these analyses will require four parameters to be estimated in the logistic regression. If there are fewer than 40 patients with and/or fewer than 40 patients without the composite endpoint BPD-free survival at 36 weeks PMA, these analyses will not be performed.

##### Sensitivity analyses

The internationally accepted definition of the primary outcome BPD is under debate, given the uncertainty on how to rate continuous positive airway pressure (CPAP) or high-flow nasal cannula (HFNC) with >2 L/minute flow and with low or no supplemental oxygen [36]. According to the international criteria used, infants who need this respiratory support should be rated as having severe BPD [31]. However, the reason for this support in these very premature infants could be an impaired control of breathing instead of chronic parenchymal lung damage. Therefore, an auxiliary analysis will be performed in which infants on CPAP and HFNC in room air will be classified as having mild BPD. If possible, we will perform sensitivity analyses on the primary outcome to investigate the impact of correlation between infant outcomes within twin or higher-order multiple births using generalised estimating equations with a logit link function.

##### Short-term secondary outcomes

The effect of HC on short-term secondary outcomes will be analysed using linear regression, logistic regression, competing risk models (that consider death before the event of interest as a competing risk) or Cox regression models, as appropriate. The time to event will be calculated as the time between randomisation and the event, death, or discharge to home, whichever occurs first. If required, non-normally distributed continuous variables will be appropriately transformed. Only a small number of SUSARs and SAEs are expected. Hence, these will be evaluated by tabulations of counts and percentages for each treatment group, respectively, and presented as absolute risk differences. These outcomes, although all pre-specified, should be considered exploratory, yielding hypothesis-generating findings, and so no formal adjustments for stratification or multiple comparisons will be made.

#### Long-term outcomes at 2 years corrected age (study phase II)

##### Neurodevelopmental impairment

The key long-term outcome is survival without neurodevelopmental impairment at 2 years CA. Participants who are alive and have a BSID III cognitive and motor composite score at 2 years CA ≥ 85 will be defined as having survived without neurodevelopmental impairment. If no BSID III test could be done because of impairment, the attending paediatrician was asked to fill in an estimate of cognitive delay in three categories: no delay, 3–6 months delay or > 6 months delay. A delay of ≥ 3 months is considered neurodevelopmental impairment and equivalent to a BSID III cognitive and motor composite score < 85. Parents of participants who do not attend the 2-year assessment at the outpatient clinic will be invited by telephone once more. If they refuse to attend, the reason for refusal will be documented. On the basis of available information about their neurological and developmental (ab)normality, participants will be classified as having neurodevelopmental impairment or not, if reasonably possible. If there is insufficient information to classify the neurodevelopmental outcome in > 5% of the participants, missing continuous BSID III scores will be imputed using multiple imputation using baseline characteristics. As previously recommended [37], we will obtain a number of imputed datasets equal to the percentage of missing data. These datasets will be combined using Rubin’s rules [37, 38]. Inspection and imputation of missing data will be performed during the blinded review of the data. This strategy will be updated if, during the blinded data review, unexpected patterns are detected, requiring an appropriately adapted handling procedure. In that case, relevant deviations will be clearly documented and justified. Statistical analysis will be performed in the same way as for the short-term primary outcome. If multiple imputation is performed, three additional sensitivity analyses will be performed to test the robustness of the results obtained. These sensitivity analyses will be performed (a) using complete cases only and by applying (b) best case and (c) worst case scenarios for the unobserved neurodevelopmental impairment outcome data.

##### Other long-term secondary outcomes

The effect of HC on the other long-term secondary outcomes will be analysed using linear, logistic or Cox regression or competing risks models, as appropriate. The time to event will be calculated as the time between randomisation and the event, death, or 2 years CA, whichever occurs first. If required, non-normally distributed continuous variables will be appropriately transformed. These outcomes, although all pre-specified, should be considered exploratory, yielding hypothesis-generating findings, and so no formal adjustments for stratification or multiple comparisons will be made.

#### Differences between the study protocol and statistical analysis plan

We added the AT analysis, thus creating a group that did not receive corticosteroids and a group that did receive corticosteroids, to check the robustness of the main analyses in the ITT population. We clarified that the interim analyses were performed solely to highlight safety issues and that there was no intention to stop the trial early for efficacy. This was to maintain power for the neurodevelopmental outcome at 2 years CA. We also clarified how the analyses would be performed and how stratification factors would be included in the analyses.

### Trial status

Initially, 15 participating centres started recruitment between December 2011 and July 2012, and 4 additional centres started recruitment in 2015. Recruitment stopped on 31 December 2016, and the last assessment of the primary outcome was completed by March 2017. The last follow-up at 2 years CA is expected around March 2019. Before the data are analysed, the database will be cleaned and checked for completeness and internal consistency, blinded to treatment allocation. The database will be locked only after this SAP has been submitted for publication in a peer-reviewed journal.

## Abbreviations

ARR: Absolute risk reduction
AT: As treated
BPD: Bronchopulmonary dysplasia
BSID III: Bayley Scales of Infant and Toddler Development, Third Edition
CA: Corrected age
CONSORT: Consolidated Standards of Reporting Trials
CPAP: Continuous positive airway pressure
DSMB: Data and safety monitoring board
FiO_2_: Fraction of inspired oxygen
GCP: Good clinical practice
HC: Hydrocortisone
HFNC: High-flow nasal cannula
ITT: Intention to treat
IVH: Intraventricular haemorrhage
MAwP: Mean airway pressure
NEC: Necrotising enterocolitis
NICU: Neonatal intensive care unit
NNT: Number needed to treat
PMA: Postmenstrual age
PNA: Postnatal age
PP: Per protocol
PVL: Periventricular leucomalacia
RCT: Randomised controlled trial
RI: Respiratory index
ROP: Retinopathy of prematurity
SAE: Serious adverse event
SAP: Statistical analysis plan
SIP: Spontaneous intestinal perforation
SToP-BPD study: Systemic Hydrocortisone To Prevent Bronchopulmonary Dysplasia in preterm infants study
SUSAR: Suspected unexpected serious adverse drug reaction

## References

[1] Horbar JD, Carpenter JH, Badger GJ, Kenny MJ, Soll RF, Morrow KA, Buzas JS (2012). Mortality and neonatal morbidity among infants 501 to 1500 grams from 2000 to 2009. Pediatrics..

[2] Shah PS, Lui K, Sjörs G, Mirea L, Reichman B, Adams M, Modi N, Darlow BA, Kusuda S, San Feliciano L (2016). Neonatal outcomes of very low birth weight and very preterm neonates: an international comparison. J Pediatr.

[3] Lapcharoensap W, Gage SC, Kan P, Profit J, Shaw GM, Gould JB, Stevenson DK, O’Brodovich H, Lee HC (2015). Hospital variation and risk factors for bronchopulmonary dysplasia in a population-based cohort. JAMA Pediatr..

[4] Jobe AH, Bancalari E (2001). Bronchopulmonary dysplasia. Am J Respir Crit Care Med..

[5] Doyle LW, Ehrenkranz RA, Halliday HL (2014). Early (< 8 days) postnatal corticosteroids for preventing chronic lung disease in preterm infants. Cochrane Database Syst Rev..

[6] Doyle LW, Ehrenkranz RA, Halliday HL (2014). Late (> 7 days) postnatal corticosteroids for chronic lung disease in preterm infants. Cochrane Database Syst Rev..

[7] American Academy of Pediatrics Committee on Fetus and Newborn (2002). Canadian Paediatric Society Fetus and Newborn Committee. Postnatal corticosteroids to treat or prevent chronic lung disease in preterm infants. Pediatrics..

[8] American Academy of Pediatrics Committee on Fetus and Newborn (2010). Policy statement—postnatal corticosteroids to prevent or treat bronchopulmonary dysplasia. Pediatrics..

[9] Onland W, Offringa M, Cools F, De Jaegere AP, Rademaker K, Blom H, Cavatorta E, Debeer A, Dijk PH, van Heijst AF (2011). Systemic Hydrocortisone To Prevent Bronchopulmonary Dysplasia in preterm infants (the SToP-BPD study); a multicenter randomized placebo controlled trial. BMC Pediatr..

[10] World Medical Association (WMA). WMA Declaration of Helsinki – ethical principles for medical research involving human subjects. https://www.wma.net/policies-post/wma-declaration-of-helsinki-ethical-principles-for-medical-research-involving-human-subjects/. 19886379

[11] Wet medisch-wetenschappelijk onderzoek met mensen http://wetten.overheid.nl/BWBR0009408/2017-03-01.

[12] Act of 26 February 1998, containing rules on medical research involving human subjects (Medical Research (Human Subjects) Act). http://www.ccmo.nl/attachments/files/wmo-engelse-vertaling-29-7-2013-afkomstig-van-vws.pdf.

[13] De experimentenwet (7 mei 2004) en Uitvoeringsbesluit KB (koninklijk besluit) (30 juni 2004) https://www.fagg-afmps.be/en/human_use/medicines/medicines/research_development/clinical_trials.

[14] International Conference on Harmonisation of Technical Requirements for Registration of Pharmaceuticals for Human Use (ICH). ICH harmonised tripartite guideline for good clinical practice E6(R1). 10 Jun 1996. http://www.ich.org/fileadmin/Public_Web_Site/ICH_Products/Guidelines/Efficacy/E6/E6_R1_Guideline.pdf.

[15] Kessler KM (2002). The CONSORT statement: explanation and elaboration. Ann Intern Med..

[16] Walsh MC, Wilson-Costello D, Zadell A, Newman N, Fanaroff A (2003). Safety, reliability, and validity of a physiologic definition of bronchopulmonary dysplasia. J Perinatol..

[17] Walsh MC, Yao Q, Gettner P, Hale E, Collins M, Hensman A, Everette R, Peters N, Miller N, Muran G (2004). Impact of a physiologic definition on bronchopulmonary dysplasia rates. Pediatrics..

[18] Neu J, Walker WA (2011). Necrotizing enterocolitis. N Engl J Med..

[19] Ment LR, Bada HS, Barnes P, Grant PE, Hirtz D, Papile LA, Pinto-Martin J, Rivkin M, Slovis TL (2002). Practice parameter: neuroimaging of the neonate: report of the Quality Standards Subcommittee of the American Academy of Neurology and the Practice Committee of the Child Neurology Society. Neurology..

[20] International Committee for the Classification of Retinopathy of Prematurity (2005). The International Classification of Retinopathy of Prematurity revisited. Arch Ophthalmol..

[21] Bayley N (2006). Bayley Scales of Infant Development, Third Edition, Manual.

[22] Palisano R, Rosenbaum P, Walter S, Russell D, Wood E, Galuppi B (1997). Development and reliability of a system to classify gross motor function in children with cerebral palsy. Dev Med Child Neurol..

[23] Achenbach TM, Ruffle TM (2000). The Child Behavior Checklist and related forms for assessing behavioral/emotional problems and competencies. Pediatr Rev..

[24] Schmidt B, Roberts R, Millar D, Kirpalani H (2008). Evidence-based neonatal drug therapy for prevention of bronchopulmonary dysplasia in very-low-birth-weight infants. Neonatology..

[25] Onland W, De Jaegere APMC, Offringa M, van Kaam AHLC (2008). Effects of a higher versus a lower dexamethasone dose on pulmonary and neurodevelopmental sequelae in preterm infants at risk for chronic lung disease: a meta-analysis. Pediatrics..

[26] Onland W, Offringa M, De Jaegere AP, van Kaam AH (2009). Finding the optimal postnatal dexamethasone regimen for preterm infants at risk of bronchopulmonary dysplasia: a systematic review of placebo-controlled trials. Pediatrics..

[27] Onland W, van Kaam AH, De Jaegere AP, Offringa M (2010). Open-label glucocorticoids modulate dexamethasone trial results in preterm infants. Pediatrics..

[28] van Rossem MC, van de Loo M, Laan BJ, de Sonnaville ES, Tamminga P, van Kaam AH, Onland W (2015). Accuracy of the diagnosis of bronchopulmonary dysplasia in a referral-based health care system. J Pediatr.

[29] Kahan BC (2014). Accounting for centre-effects in multicentre trials with a binary outcome - when, why, and how?. BMC Med Res Methodol..

[30] Peduzzi P, Concato J, Kemper E, Holford TR, Feinstein AR (1996). A simulation study of the number of events per variable in logistic regression analysis. J Clin Epidemiol..

[31] Bancalari E, Claure N (2006). Definitions and diagnostic criteria for bronchopulmonary dysplasia. Semin Perinatol..

[32] Pugni L, Pietrasanta C, Acaia B, Merlo D, Ronchi A, Ossola MW, Bosari S, Mosca F (2016). Chorioamnionitis and neonatal outcome in preterm infants: a clinical overview. J Matern Fetal Neonatal Med..

[33] Walsh MC, Morris BH, Wrage LA, Vohr BR, Poole WK, Tyson JE, Wright LL, Ehrenkranz RA, Stoll BJ, Fanaroff AA (2005). Extremely low birthweight neonates with protracted ventilation: mortality and 18-month neurodevelopmental outcomes. J Pediatr..

[34] Fanaroff AA, Stoll BJ, Wright LL, Carlo WA, Ehrenkranz RA, Stark AR, Bauer CR, Donovan EF, Korones SB, Laptook AR (2007). Trends in neonatal morbidity and mortality for very low birthweight infants. Am J Obstet Gynecol..

[35] Yu KH, Li J, Snyder M, Shaw GM, O’Brodovich HM (2016). The genetic predisposition to bronchopulmonary dysplasia. Curr Opin Pediatr..

[36] Poindexter BB, Feng R, Schmidt B, Aschner JL, Ballard RA, Hamvas A, Reynolds AM, Shaw PA, Jobe AH (2015). Prematurity and Respiratory Outcomes Program. Comparisons and limitations of current definitions of bronchopulmonary dysplasia for the Prematurity and Respiratory Outcomes Program. Ann Am Thorac Soc..

[37] White IR, Royston P, Wood AM (2011). Multiple imputation using chained equations: issues and guidance for practice. Stat Med..

[38] Schafer JL (1999). Multiple imputation: a primer. Stat Methods Med Res..

